# Supplementary material for: Improvement in Visceral Adipose Tissue and LDL Cholesterol by High PUFA Intake: 1-Year Results of the NutriAct Trial
Source: Nutrients. 2024 Apr 4;16(7):1057. doi: 10.3390/nu16071057 (PMC11013849; doi:10.3390/nu16071057)
Supplement: Supplementary file 1 [file nutrients-16-01057-s001.zip › nutrients-2882798-supplementary.pdf]

Supplemental Material

Improvement of visceral adipose tissue and LDL cholesterol by high PUFA intake: 1-year results of the NutriAct trial

Nina Marie Tosca Meyer[1,2], Anne Pohrt[3], Charlotte Wernicke[1,2], Laura Pletsch-Borba[1,2,4], Konstantina Apostolopoulou[1,2], Linus Haberbosch[1,2,5], Jürgen Machann[6,7,8], Andreas F H Pfeiffer[1,2,8], Joachim Spranger[1,2,8-11], Knut Mai[1,2,8-11]

[1] Charité – Universitätsmedizin Berlin, corporate member of Freie Universität Berlin, Humboldt-Universität zu Berlin, and Berlin Institute of Health; Department of Endocrinology and Metabolism; Charitéplatz 1, 10117 Berlin, Berlin, Germany; nina.meyer@charite.de; charlotte.wernicke@charite.de; laura.pletsch-borba@charite.de; linus.haberbosch@charite.de; andreas.pfeiffer@charite.de; joachim.spranger@charite.de; knut.mai@charite.de

[2] NutriAct-Competence Cluster Nutrition Research Berlin-Potsdam, Arthur-Scheunert-Allee 114 - 116, 14558 Nuthetal Germany

[3] Charité – Universitätsmedizin Berlin, corporate member of Freie Universität Berlin, Humboldt-Universität zu Berlin, and Berlin Institute of Health, Institute of Biometry and Clinical Epidemiology, Charitéplatz 1, 10117 Berlin, Berlin, Germany; anne.pohrt@charite.de

[4] Berlin Institute of Health at Charité – Universitätsmedizin Berlin, BIH Biomedical Innovation Academy, BIH Charité Junior Clinician Scientist Program, Charitéplatz 1, 10117 Berlin, Germany

[5] Berlin Institute of Health at Charité – Universitätsmedizin Berlin, BIH Biomedical Innovation Academy, BIH Charité Junior Digital Clinician Scientist Program, Charitéplatz 1, 10117 Berlin, Germany

[6] Institute for Diabetes Research and Metabolic Diseases (IDM) of the Helmholtz Center Munich at the University of Tübingen, Otfried-Müller-Str. 10

72076 Tübingen, Baden-Württemberg, Germany

[7] Section on Experimental Radiology, Department of Diagnostic and Interventional Radiology, University Hospital Tübingen, Otfried-Müller-Straße 12/1

72076 Tübingen, Baden-Württemberg, Germany; juergen.machann@med.uni-tuebingen.de

[8] German Center for Diabetes Research, Ingolstaedter Landstrasse 1, 85764 München-Neuherberg, Bayern, Germany

[9] Department of Human Nutrition, German Institute of Human Nutrition, Potsdam-Rehbruecke, Arthur-Scheunert-Allee 114-116, 14558 Nuthetal, Brandenburg, Germany

[10] DZHK (German Centre for Cardiovascular Research), partner site Berlin, Potsdamer Str. 58, 10785 Berlin, Berlin, Germany

[11] Max Rubner Center for Cardiovascular Metabolic Renal Research, Hessische Str. 3-4, 10115 Berlin, Berlin, Germany

Corresponding author: Prof. Knut Mai, Universitätsmedizin Berlin, Department of Endocrinology and Metabolism, corporate member of Freie Universität Berlin, Humboldt-Universität zu Berlin, and Berlin Institute of Health, Charitéplatz 1, 10117 Berlin, Tel: +49/30/450-514252, Fax: +49/30/450-514950, e-mail: knut.mai@charite.de

Table of Contents

1.	Details on inclusion and exclusion criteria	p. 3
2.	Details on sample size calculation	p. 3
3.	Details on randomization process	p. 3
4.	Details on quantification of intrahepatic lipids	p. 4
5.	Dietary Intervention	p. 4
6.	Table S1	p. 4
7.	Table S2	p. 5
8.	Tables S3 a + b	p. 6
9.	Tables S4 a + b	p. 7
10.	Figure S1	p. 8
11.	References	p. 9

1. Details on inclusion and exclusion criteria

Inclusion criteria

Presence of at least one of the following pre-specified features of unhealthy aging, defined as:

- arterial hypertension: systolic blood pressure ≥ 140 mmHg/ diastolic blood pressure ≥ 90 mmHg/ positive history for hypertension/ intake of antihypertensive medication
- heart failure: New York Heart Association ≥II or N-terminal pro-B-type natriuretic peptide >300ng/l without atrial fibrillation
- existent cardiovascular disease: previous myocardial infarction or stroke/ coronary heart disease/ peripheral artery disease
- evidence of cognitive dysfunction: Montreal Cognitive Assessment Score <26
- decline in muscular strength: Short Physical Performance Battery Score <10

Exclusion criteria

- concerning severe kidney disease: defined as: GFR < 50 ml/min/1.73m²

2. Details on sample size calculation

We conducted a sample size calculation based on the primary endpoint of the NutriAct trial for a log-rank test. The primary outcome of the NutriAct trial is a composite endpoint of age-related disorders (encompassing cardiovascular morbidity, decline of cognitive function and clinical features of sarcopenia). A power of 80% was selected with a two-sided alpha of 5%. The anticipated hazard ratios varied between 1.42 and 2.11. Consequently, sample sizes ranging from 200 to 226 participants were determined for each group. To account for an estimated 10-15% dropout rate, we aimed to recruit 502 participants initially. The calculations were carried out using nQuery Advisor V7..

3. Details on randomization process

As specified in (1), randomization was done in a stratified manner using pre-specified characteristics - which were sex, presence or no presence of CVD, heart failure (NYHA ≥ 2 or NT-pro-BNP > 300mg/l if no atrial fibrillation), arterial hypertension, type 2 diabetes, impaired cognitive (MoCA < 26) and physical function (SPPB < 10) - with additional adaptive randomization (1:1). Family members or individuals residing in the same household were assigned to the same group. Assignments were announced after baseline phenotyping, guaranteeing concealment of allocation. A blinding of participants was not feasible owing to the type of intervention. Trained study personnel conducted all examinations following standard operating procedures. Pseudonymization was applied on data acquisition and analysis.

4. **Details on quantification of intrahepatic lipids via MR spectroscopy (1H-MRS)**

For quantification of IHL, a single voxel stimulated echo acquisition mode (STEAM) technique was applied with a voxel (volume of interest) size of 30x30x20mm³ in the posterior part of segment 7 with a short echo time (TE= 10 ms) and a long repetition time (TR= 4 s) to minimize relaxation-based bias(2). Integral of fat (methylene+methyl resonances at 1.3 and .9 ppm, respectively) was divided by the sum of water (at 4.7 ppm) and fat resonances, respectively, resulting in ratios for indication of IHL.

5. **Dietary intervention**

In the intervention group, participants were asked to increase intake of foods rich in protein, fibers and/or PUFA. More specifically, main recommendations were as follows: daily intake of rapeseed oil: 20 g for women, 30g for men, flaxseed meal: 10 g, nuts or seeds (with preference of walnuts): 30 g, vegetables and salads: 400 g, fruits: 250 g, milk or dairy products: 200-250 g, cheese: 50-60 g; weekly intake of protein flakes: 40 g, protein rolls: 4, protein noodles: 1 portion, legumes: 2 portions, fish: 2 portions, low fat meat and sausages: 300-600 g.
In order to meet the recommended NutriAct pattern, participants were provided with foods free of charge that were specifically designed in accordance with the predefined dietary pattern. Supplementary Table 1 illustrates the food products used within the first year of intervention.

In the control group, participants were asked to follow a balanced health-promoting diet as recommended by the German Nutrition Society (3, 4). Overall, participants were asked to reduce daily intake of total fat and SFA as well as sugar. Specific recommendations for daily intake included: cereal products: 4-6 portions (200 – 250 g) with preference from whole grains, potatoes/ rice/ noodles: 1 portion, vegetables and salad: 3 portions (400 g), fruits: 2 portions (250 g), milk or dairy products: 200-250 g, cheese: 50-60 g, with preference from low fat products, oil: 10-15 g, spreadable fat: 15-30 g, calorie-free or low-calorie beverages: circa 1,5 L. Additional recommendations for weekly intake were: low fat meat and sausages: 300-600 g, fish: 2 portions, eggs: max. 3.

Throughout the entire study period, participants of the control group were provided with 1 package of high carbohydrate muesli (435 g), 2 packages of barley flakes (250 g) and a package of raw barley as an alternative to rice (250 g), respectively.

These instructions were given within nutritional counselling sessions. These approx. 2-hour sessions were held in closed groups of 6-10 participants. The individual topics of these consultations were designed to support the participants in changing their dietary habits according to the above mentioned requirements.

6. **Table S1. Composition of the provided NutriAct foods**

	Protein flakes	Protein pasta	Rolls with a topping of sunflower seeds	Rolls with a topping of pumpkin seeds	Flaxseed meal	Rapeseed oil
**Energy [kJ/kcal][1]**	1700/ 402	1398/ 330	998/ 239	1028/ 247	1528/ 369	3404/ 828
**Protein [g][1]**	59.5	23.0	14.9	15.7	31.7	0
**Carbohydrates [g][1]**	25.2	52.5	22	20	2.7	0
of which sugar [g]	9.4	3.6	1.8	1.7	2.7	0
**Fat [g][1]**	6.4	1.1	8.0	9.4	18.8	92.0
of which SFA [g]	1.4	0.2	1.2	1.6	2.0	6.5
of which PUFA [g]	3.1	0.7	n/a	n/a	12.6	25.5
of which MUFA [g]	1.9	0.2	n/a	n/a	2.9	60.0

**Dietary fiber [g]**[1]	2.9	9.2	8.4	8.6	30.9	0
**Salt [g]**[1]	1.6	0.6	1.0	1.0	0	0

[1]per 100g, for rapeseed oil per 100 ml; SFA, saturated fatty acids; PUFA, polyunsaturated fatty acids; MUFA, monounsaturated fatty acids, n/a = not available

7. Table S2. Baseline correlations between VAT and anthropometric and metabolic parameters

**Baseline characteristics**	**Pearson correlation coefficient**	**P-value for the correlation with baseline VAT**	**N**
BMI	.635	**<.001**	300
Waist circumference	.682	**<.001**	300
HOMA-IR	.502	**<.001**	299
TC (mmol/l)	-.18	**.002**	300
LDL-C (mmol/l)	-.118	**.042**	299
HDL-C (mmol/l)	-.453	**<.001**	300
TG (mmol/l)	.258	**<.001**	300
Protein intake ($g*kg^{-1}*d^{-1}$)	-.272	**<.001**	293
Carbohydrate intake (% $E*d^{-1}$)	-.170	**.004**	293
Fiber intake ($g*d^{-1}$)	-.115	.050	293
MUFA intake (%$E*d^{-1}$)	.057	.333	293
PUFA intake (%$E*d^{-1}$)	-.027	.649	293
SFA (%$E*d^{-1}$)	.048	.409	293

Pearson Correlation Analysis. Dietary components are described as % from the total daily energy intake, except for protein and fiber intake, which are described as $g*kg^{-1}$ bodyweight and $g*d^{-1}$ respectively. The significant association between VAT and TC as well as LDL-C were diminished after adjustment for lipid-lowering medication, respectively (TC: ρ= -.84, p= .151; LDL-C: ρ= -.005, p= .933). The other significances stayed stable after adjustment for antidiabetic (HOMA-IR) or lipid-lowering (HDL, Tg) medication. Abbreviations: BMI body mass index, HOMA-IR Homeostatic model assessment-Insulin resistance, TC total cholesterol, LDL-C low density lipoprotein cholesterol, HDL-C high density lipoprotein cholesterol, TG triacylglycerols, MUFA monounsaturated fatty acids, PUFA polyunsaturated fatty acids, SFA saturated fatty acids.

8. Tables S3a + b. Correlations between changes in VAT/ NVAT and macronutrients

a. Δ VAT

Δ **VAT**	**Pearson correlation coefficient**	**P-value**	**N**
Δ Protein intake ($g*kg^{-1}*d^{-1}$)	-.116	**.049**	290
Δ Fiber intake ($g*d^{-1}$)	-.103	.080	290
Δ Carbohydrates intake (% E*d-1)	.050	.401	290
Δ MUFA intake ($\%E*d^{-1}$)	.048	.416	290
Δ PUFA intake ($\%E*d^{-1}$)	-.177	**.003**	290
Δ SFA intake ($\%E*d^{-1}$)	.099	.093	290

Pearson Correlation Analysis. Dietary components are described as % from the total daily energy intake, except for protein and fiber intake, which are described as $g*kg^{-1}$ bodyweight and $g*d^{-1}$ respectively. Abbreviations: VAT visceral adipose tissue, MUFA monounsaturated fatty acids, PUFA polyunsaturated fatty acids, SFA saturated fatty acids. Δ Delta.

b. Δ NVAT

Δ **NVAT**	**Pearson correlation coefficient**	**P-value**	**N**
Δ Protein intake ($g*kg^{-1}*d^{-1}$)	-.090	.129	285
Δ Fiber intake ($g*d^{-1}$)	-.056	.343	285
Δ Carbohydrates intake (% E*d-1)	.086	.147	285
Δ MUFA intake ($\%E*d^{-1}$)	-.009	.876	285
Δ PUFA intake ($\%E*d^{-1}$)	-.227	**<.001**	285
Δ SFA intake ($\%E*d^{-1}$)	.101	.088	285

Pearson Correlation Analysis. Dietary components are described as % from the total daily energy intake, except for protein and fiber intake, which are described as $g*kg^{-1}$ bodyweight and $g*d^{-1}$ respectively. Abbreviations: NVAT non-visceral adipose tissue MUFA monounsaturated fatty acids, PUFA polyunsaturated fatty acids, SFA saturated fatty acids. Δ Delta.

9. Tables S4 a + b. Mediation analyses

a. Intervention – ∆PUFA – ∆VAT – ∆LDL

Variables			B	Std. Error	P-value
Intervention Group	→	∆ PUFA intake	3.428	.448	<.001
∆ PUFA intake	→	∆ VAT	-.028	.010	.006
Intervention Group	→	∆ VAT	-.063	.083	.443
∆ VAT	→	∆ LDL-C	.116	.059	.047
∆ PUFA intake	→	∆ LDL-C	-.013	.011	.241
Intervention Group	→	∆ LDL-C	-.174	.087	.046

Adjusted for baseline LDL, baseline VAT, age, sex and change in lipid-lowering medication.

b. Intervention – ∆PUFA – ∆NVAT – ∆LDL

Variables			B	Std. Error	P-value
Intervention Group	→	∆ PUFA intake	3.428	.448	<.001
∆ PUFA intake	→	∆ NVAT	-.063	.018	<.001
Intervention Group	→	∆ NVAT	-.196	.152	.197
∆ NVAT	→	∆ LDL-C	.048	.031	.131
∆ PUFA intake	→	∆ LDL-C	-.013	.011	.225
Intervention Group	→	∆ LDL-C	-.167	.088	.057

Adjusted for baseline LDL, baseline VAT, age, sex and change in lipid-lowering medication.

10. Figure S1. CONSORT 2010 Flow Diagram of the Study Design, VAT cohort

CONSORT
TRANSPARENT REPORTING of TRIALS

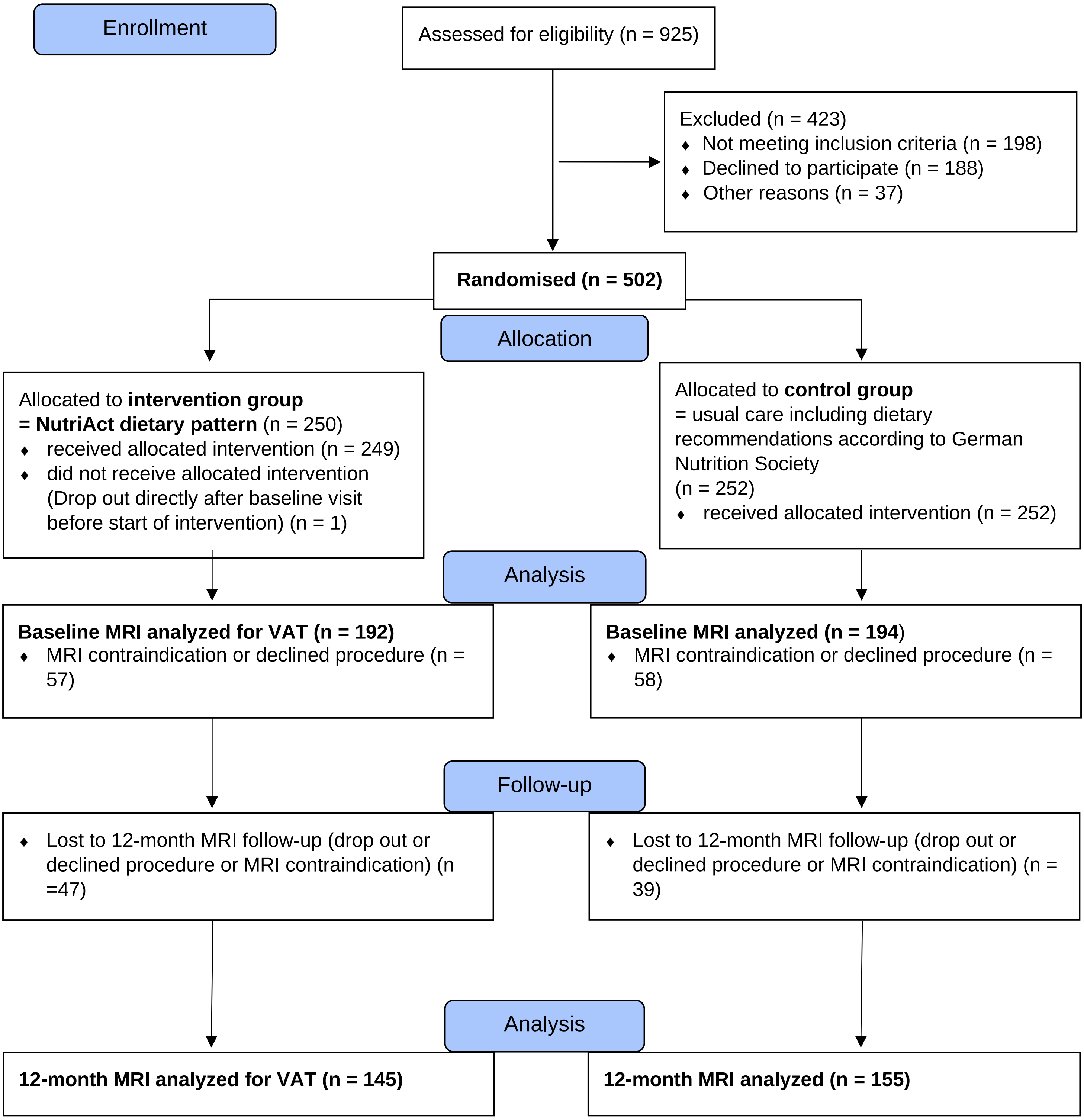

11. References

1. Wernicke C, Apostolopoulou K, Hornemann S, Efthymiou A, Machann J, Schmidt S, et al. Long-term effects of a food pattern on cardiovascular risk factors and age-related changes of muscular and cognitive function. Medicine. 2020;99(39):e22381-e.
2. Machann J, Thamer C, Stefan N, Schwenzer NF, Kantartzis K, Häring HU, et al. Follow-up whole-body assessment of adipose tissue compartments during a lifestyle intervention in a large cohort at increased risk for type 2 diabetes. Radiology. 2010;257(2):353-63.
3. Wolfram G, Bechthold A, Boeing H, Ellinger S, Hauner H, Kroke A, et al. Evidence-Based Guideline of the German Nutrition Society: Fat Intake and Prevention of Selected Nutrition-Related Diseases. Annals of nutrition & metabolism. 2015;67(3):141-204.
4. Deutsche Gesellschaft für Ernährung (DGE), Österreichische Gesellschaft für Ernährung (ÖGE), Schweizerische Gesellschaft für Ernährung (SGE). Referenzwerte für die Nährstoffzufuhr2015.
